# Mesoscale Mechanisms in Viscoplastic Deformation of Metals and Their Applications to Constitutive Models

**DOI:** 10.3390/ma14164667

**Published:** 2021-08-19

**Authors:** Wen Lai Huang, Lin Zhang, Kaiguo Chen, Guo Lu

**Affiliations:** 1 State Key Laboratory of Multiphase Complex Systems, Institute of Process Engineering, Chinese Academy of Sciences, Beijing 100190, China; zhanglin@ipe.ac.cn; 2 National Key Laboratory of Shockwave Physics and Detonation Physics, Institute of Fluid Physics, Chinese Academy of Engineering Physics, Mianyang 621900, China; chenkaiguo@caep.cn; 3 Laboratory of Computational Physics, Institute of Applied Physics and Computational Mathematics, Beijing 100088, China; lu_guo@iapcm.ac.cn

**Keywords:** dislocation, shock wave, viscoplastic deformation, constitutive model, heterogeneity, mesoscale

## Abstract

Deformation of metals has attracted great interest for a long time. However, the constitutive models for viscoplastic deformation at high strain rates are still under intensive development, and more physical mechanisms are expected to be involved. In this work, we employ the newly-proposed methodology of mesoscience to identify the mechanisms governing the mesoscale complexity of collective dislocations, and then apply them to improving constitutive models. Through analyzing the competing effects of various processes on the mesoscale behavior, we have recognized two competing mechanisms governing the mesoscale complex behavior of dislocations, i.e., maximization of the rate of plastic work, and minimization of the elastic energy. Relevant understandings have also been discussed. Extremal expressions have been proposed for these two mesoscale mechanisms, respectively, and a stability condition for mesoscale structures has been established through a recently-proposed mathematical technique, considering the compromise between the two competing mechanisms. Such a stability condition, as an additional constraint, has been employed subsequently to close a two-phase model mimicking the practical dislocation cells, and thus to take into account the heterogeneous distributions of dislocations. This scheme has been exemplified in three increasingly complicated constitutive models, and improves the agreements of their results with experimental ones.

## 1. Introduction

Understanding the viscoplastic behavior of metals at high strain rates is of critical importance in many significant fields [1,2], and developing effective Crystal Plasticity (CP) constitutive models has been a continuous effort for decades [2,3]. Constitutive models describe the relationship between the stress *σ* and the strain *ε* or the strain rate $\dot{\varepsilon }$, and dislocations (in normal cases) are the key media in between, in the cases where twinning and phase transformation (these two aspects can be significant in special cases) are insignificant. A constitutive model usually consists of three parts: Kinematics (describing the relationship between $\dot{\varepsilon }$ and dislocation density *N* and speed *v*, e.g., the Orowan relation or its generalized form [4]), kinetics (describing the relationship between *σ* and *N* and *v*), and the time evolution of *N*. As for the frequently encountered models, the Johnson-Cook (JC) [5,6] and Steinberg-Guinan (SG) [7,8] models do not employ dislocations directly, while Zerilli-Armstrong (ZA) [9,10], Mechanical Threshold Stress (MTS) [11,12], and Preston-Tonks-Wallace (PTW) [13] models utilize dislocations implicitly, and the Livermore Multiscale Strength (LMS) [14] and Austin-McDowell (AM) [2] models adopt dislocations explicitly. Obviously, information of dislocations has been involved more and more directly and explicitly, reflecting the increasingly improved understandings on relevant physical mechanisms.

However, two aspects of limitations are still apparent, indicating the insufficiency of our understandings. Firstly, lots of fitting parameters have been used in constitutive models, which are empirical and poorly transferable. Secondly, heterogeneous structures have not been well accounted for, and thus size effects have hardly been addressed [15]. This means that more physical mechanisms are still needed to provide more bases or constraints for the models. To this end, the emerging methodology of mesoscience [16,17] is helpful.

Based on decades of efforts in modeling several important complex systems in chemical engineering, mesoscience was proposed and gradually enriched recently [16,17,18,19,20]. In this methodology, for a system with massive elements, the length scale of an element is called the element scale, the length scale of the system is called the system scale, both of which are called boundary scales, and the length scale between the element scale and the system scale (i.e., two adjacent boundary scales) is called the mesoscale scale. These scales are all dependent on the system of interest, and thus are relative scales, not absolute sizes. The element scale, the mesoscale, and the system scale of a system cover a scale level (called level for brevity). At a level, different systems might exist, and at different levels, different systems certainly exist. For two adjacent levels, the system scale at the lower level is the element scale at the higher level.

The starting point in mesoscience begins when modeling a complex system, the mesoscale heterogeneous structures should be accounted for, and the mean-field approximation fails. Meanwhile, we need not and usually cannot rely on all the information at the element scale (the behavior of all elements), but reveal and thus employ the specific laws at the mesoscale [21]. Accumulated evidence reveals that mesoscale complex structures can be described with the compromise between different competing extremal mechanisms (called dominant mechanisms). Since mesoscale complex structures emerge only under specific conditions, i.e., within specific regimes (called mesoregimes [19]), there is no complexity in the adjacent trivial regimes (called extreme regimes). Importantly, it is increasingly clear that there only one dominant mechanism might exist in each extreme regime, and all the dominant mechanisms in adjacent extreme regimes will probably constitute the multiple competing dominant mechanisms for the in-between mesoregime. The Compromise in Competition (CIC) of different dominant mechanisms is expressed as a multi-objective optimization (or variational) problem, and solving it usually needs transforming it further into a single-objective one (called the stability condition of the mesoscale complex structures). The expressions of dominant mechanisms are system-specific and thus level-specific, thus the proper resolution of levels and systems is necessary for the identification of dominant mechanisms [17]. These points will be employed to analyze the metals deformation here.

The whole work will be presented in the following sequence: In Section 2, we will distinguish the levels of complexity involved in metals deformation, and point out that the present focus is on the level corresponding to the collective behavior of dislocations. In Section 3, we will analyze the involved mesoscale structures at the focused level, and classify relevant processes into competing groups, according to their different influences on the mesoscale structures. In Section 4, two dominant mechanisms governing the mesoscale structures are proposed within the mesoscience framework, based on the preceding analysis on the competing tendencies, and their competition and compromise are described. In Section 5, we apply the dominant mechanisms to three increasingly complicated constitutive models to validate their significance in accounting for heterogeneous distributions of dislocations, exemplified with 6061-T6 Al alloy under shock stress amplitudes of 2.1, 3.7, and 9.0 GPa, respectively. Model #1 is typical but simple, taken directly from that of Molinari and Ravichandran [22]. Model #2 is based on the framework of Molinari and Ravichandran [22], but incorporates a detailed dislocation-density evolution [2], thus it is a combined model. Model #3 considers both the complicated evolution of dislocation densities and the complicated descriptions of the dislocation movements under both thermal activation and phonon drag, taken essentially from [2], with some values of parameters borrowed from Austin and McDowell [4], as well. Finally, we propose our concluding remarks in Section 6.

## 2. Two Levels of Mesoscales

**Figure 1:**
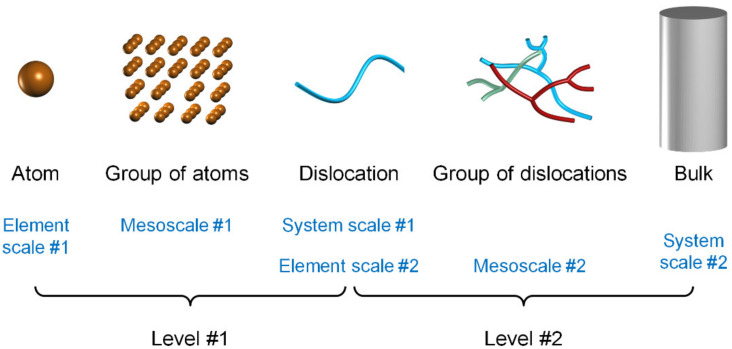
Two typical levels of mesoscale complexity in metals deformation.

To investigate the dislocation-based deformation of metals, we can start from the atomic scale or usually from the dislocation scale. For the former, an atom is an element, and our focus might be how the atomic behavior affects a dislocation, thus a dislocation can be a system, and the mesoscale structure might be the atomic distribution around the dislocation core, around a kink, around a jog or in the vicinity where the dislocation interacts with vacancies or interstitials. For the latter, a dislocation is an element, and our focus might be how the dislocation behavior affects the mechanical performance of the bulk, thus the bulk is a system, and the mesoscale structure is the dislocation distribution. These form two levels, as illustrated in Figure 1. Certainly, this is still a much simplified picture without considering impurities, grain boundaries, bulk surfaces, load distribution, etc. Otherwise, more mesoscale structures might be involved and more levels might be added.

According to such a resolution, Molecular Dynamics (MD) is a model at the element scale of the first level, as it describes the behavior of each atom. Dislocation Dynamics (DD) is a model at the system scale of the first level and at the element scale of the second level, since it describes the behavior of each dislocation. The above-mentioned constitutive models are at the system scale of the second level, describing the mechanical performance of the whole bulk. Therefore, MD, DD, and CP constitutive models (normally called microscopic, mesoscopic, and macroscopic models, respectively) are all at the boundary scales and the mesoscale information hardly receives special attention though it might be involved (e.g., through ways of hierarchical correlation).

It is necessary to decouple the two levels of mesoscale complexity during mesoscale modeling since dominant mechanisms are level-specific. Fortunately, the first level is almost clear already, and thus the focus can be moved solely onto the second one. In fact, when the United States boosted wide discussions on mesoscale issues in 2012 [23], the first “Mastering Defect Mesostructure and its Evolution” of the proposed six priority research directions in a detailed report [24] is actually the above second level of complexity, signifying its well-recognized importance. This level will be our topic hereinafter.

## 3. Mesoscale Structures and Relevant Processes

To reveal the mechanisms governing the mesoscale behavior, we need to identify the mesoscale structures first, and then analyze the processes producing the mesoscale structures, and the conditions affecting the processes.

Focusing on the above-mentioned second level, the mesoscale structure is the complex distribution of dislocations. Large-scale MD simulations have revealed the dramatically heterogeneous distributions of dislocations (and their velocities) [25]. Extensive experimental results have also shown structured distributions of dislocations [26,27]. The typical distributions of dislocations are dislocation cells [28] (with cell walls of high dislocation density and cell blocks of low dislocation density [29]) though certain variations exist [30,31]. The formation and evolution of such cells are concerned with many dislocation processes, including nucleation (homogeneous and heterogeneous), movement (glide and climb), changes (in length, shape, and type), reactions, disappearance on surfaces, etc. Such processes determine the dislocation density and distribution. Conditions affecting these processes include temperature, pressure, stress, strain, time, etc. Such conditions affect the dislocation processes and thus the dislocation densities and distributions (and the cell characteristics [28]).

According to the thermally-activated glide of dislocations, as expressed in Equations (1)–(3) [2] (where *v*_w_ is the average speed of overcoming an obstacle, *L* is the average distance between obstacles, *t*_w_ is the average waiting time, *ν*_G_ is the attempt frequency, Δ*G* is the free enthalpy of activation, *k* is the Boltzmann constant, *θ* is the absolute temperature, *μ* is the shear modulus, *τ* is the shear stress, *b* is the magnitude of the Burgers vector, *τ*_0_ is the mechanical threshold stress, and *g*_0_, *q*, and *r* are fitting parameters), long time, high temperature, large stress, and low *μ* (via, e.g., low pressure) will lead to more times of overcoming obstacles, and thus the long average glide distance (i.e., large strain) that leads to large probability of multiplication and homogeneous distribution under the applied definitely-directional external stress, and more structured distribution of dislocations with lower stored elastic energy under the complex internal stress of the dislocations (climb might be necessary here, but it is thermally activated as well, and the tendency might be similar).

(1) $$v_{w}=\frac{L}{t_{w}}$$

(2) $$t_{w}=\frac{1}{\nu_{G}}\left[\exp\left(\frac{\Delta G}{k\theta }\right)-1\right]$$

(3) $$\Delta G=g_{0}\mu b^{3}{\left[1-{\left(\frac{\tau }{\tau_{0}}\right)}^{q}\right]}^{r}$$

Therefore, low external stress or its strain, high internal stress, high temperature, low pressure, and long-time facilitate the reduction in the dislocation density and the elastic energy (through enhancing the extent of structurization and ordering), while high external stress or its strain, low internal stress, high temperature, low pressure, and long-time tend to increase the dislocation density and promote the homogenization and disordering of the dislocation distribution. Reduction in the dislocation density and ordering of the dislocation distribution correspond to the release of the stored elastic energy, and the relaxation to equilibrium, where principally the complex internal stress (due to the existence of dislocations) does work and the elastic energy transforms into heat. The increase in the dislocation density and disordering of the dislocation distribution correspond to the deviation from the equilibrium, where basically the definitely-directional external stress does work and the input energy transforms into heat and elastic energy. The internal elastic stress depends on the dislocation density and distribution (the local magnitudes and directions of the internal stress depend mainly on the local dislocation density), with the local magnitude increasing roughly with the local dislocation density (the Taylor relation (Equation (4)) is a preliminary guide, where *μ*_0_ is the shear modulus at 0 K, and *α*_0_ is a fitting parameter), and the directions are usually spatiotemporally different. On the contrary, the external stress might have definite magnitudes in definite directions.

(4) $$\tau_{0}=\alpha_{0}\mu_{0}b\sqrt{N}$$

In other words, the effects of temperature, pressure, and time on the dislocation movement are independent of what stress (external or internal) drives the movement. Therefore, these factors show no tendency in changing the dislocation density and distribution (favoring neither homogenization (disordering) nor structurization (ordering) of the dislocation distribution, and neither increase nor decrease in the dislocation density) though taking proper values of them might be necessary to the realization of certain tendencies (called allowable conditions hereinafter). The different tendencies of the dislocation density and distribution originate from the relative dominance between the external stress and the internal stress. When the external stress is much higher than the internal stress, the dislocation behavior will be dominated by the external stress, tending to homogenize the dislocation distribution and enhance the dislocation density. The increase in the dislocation density will improve the internal stress, and thus the tendency of ordering of the dislocation distribution and decreasing of the dislocation density. When the internal stress is much higher than the external stress and dominates the dislocation behavior, under allowable conditions, ordering and decreasing of dislocations will be dominant. Roughly speaking, during loading the overall external stress is higher than the overall internal stress, and the dislocation movement is mainly driven by the external stress. Whereas, during unloading the overall internal stress is higher than the overall external stress, and the dislocation movement is probably driven by the internal stress. However, the situation might depend on the specific loading and unloading conditions and might be much more complex locally (during loading the internal stress might drive some dislocations somewhere sometime, e.g., when the obstacle ahead of a pileup of dislocations is broken).

The extreme case where the internal stress exclusively dominates might be that the external stress is zero. This can be at the end of unloading, and the internal stress will solely drive the system to the equilibrium state of low elastic energy (strictly speaking, of low free energy but the entropy effects might be negligible due to the commonly negligible dislocation inertia and thus the negligibly low dislocation temperature). The extreme case where the external stress exclusively dominates might be approximately encountered when the applied stress is very high while the dislocation density is still very low. These constitute the two extreme regimes, and the wide in-between is the mesoregime where the external stress and the internal one coexist and both shape the dislocation density and distribution.

For the phonon-drag processes, as expressed in Equations (5)–(11) [2] (where *v*_r_ is the average glide speed between obstacles, *t*_r_ is the average glide time between obstacles, *τ*_eff_ is the effective stress, *τ_μ_* is the long-range resistance, *B* is the damping coefficient, *B*_0_ is the nominal value of *B*, *c*_s_ is the shear (or transverse) wave speed, *ρ* is the mass density, *z* is the number of atoms per unit cell, and *β*_0_ is a fitting parameter), high *τ* still leads to high *v*_r_, but high *θ* leads to large *B*_0_ and thus low *v*_r_. Large pressure causes large *μ* and thus low *τ*_eff_, leading to low *v*_r_ (when neglecting its effects on *c*_s_), which is the same trend as in thermally-activated processes. Usually the internal stress cannot reach the magnitude of driving the dislocations in the regime of phonon drag, so it is assumed that the internal stress still drive the dislocations through thermal activation when the external stress drives the dislocations in the phonon drag regime. Therefore, high external stress and low temperature will tend to increase the dislocation density and homogenize the dislocation distribution. Whereas, high internal stress and high temperature will facilitate reduction in the dislocation density and ordering of the dislocation distribution. In the phonon-drag regime, the external stress might be much higher than the internal stress, which seems to bring the dominant tendency of disordering of the dislocation distribution, but the accompanying high temperature facilitates the ordering, so the case might still lie in the mesoregime. By the way, the *z* value can also affect *B*_0_ and thus the realization of the homogenization tendency. It is 4 for face-centered cubic (fcc) metals, while only 2 for body-centered cubic (bcc) ones, which might provide another origin (in addition to the stacking fault energy) for the easier emergence of heterogeneous dislocation distributions in fcc metals than in bcc ones within the phonon-drag regime.

(5) $$v_{r}=\frac{L}{t_{r}}$$

(6) $$v_{r}=\frac{\tau_{\mathrm{eff}}b}{B}$$

(7) $$B=\frac{B_{0}}{1-{\left(v_{r}/c_{s}\right)}^{2}}$$

(8) $$B_{0}=\frac{3k\theta z}{20c_{s}b^{2}}$$

(9) $$c_{s}=\sqrt{\frac{\mu }{\rho }}$$

(10) $$\tau_{\mathrm{eff}}=\sqrt{\tau^{2}-\tau_{\mu }^{2}}$$

(11) $$\tau_{\mu }=\frac{\mu }{\mu_{0}}\beta_{0}\tau_{0}$$

## 4. Dominant Mechanisms and Their Compromise in Competition

**Figure 2:**
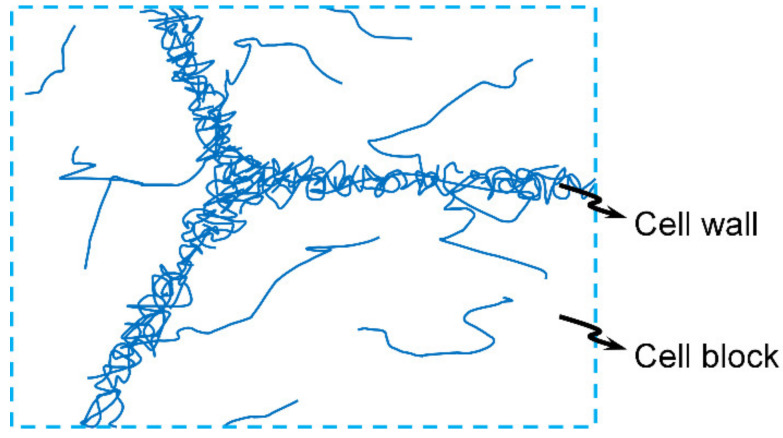
Illustration of the two-phase distribution of dislocations.

Based on the above analysis, it is clear that under either thermal activation or phonon drag, the increase in the external stress will promote the increase in the dislocation density and the disordering of the dislocation distribution. Actually, the corresponding overall process is the absorption of the input mechanical energy through the entity of dislocation due to its proper absorption spectrum (overlapping well with the input energy spectrum). To maximize the absorption, the rate of plastic work maximizes under the fixed external stress (this seems consistent with the MaxEPP proposal of Ziegler [32] since the energy dissipation rate might maximize, as well). Therefore, this first tendency of disordering and increasing of dislocations might be expressed as Equation (12) when considering a two-phase structure (as illustrated in Figure 2), where the subscript “w” means the dense wall phase, and “b” means the dilute block phase or the maximization of the rate of plastic work under the external stress, as expressed in Equation (13) for uniaxial-strain conditions (where *λ*_1_ is the longitudinal stretch, and *ϕ* is the plastic shearing rate) or the maximization of the total energy dissipation rate Σ, as in Equation (14). This is the first dominant mechanism.

(12) $$\min\frac{{\dot{N}}_{w}-{\dot{N}}_{b}}{\dot{N}}=\frac{dN_{w}-dN_{b}}{dN}$$

(13) $$\max{\dot{W}}_{p}=-\frac{4}{3}\lambda_{1}\tau \varphi$$

(14) maxΣ

The other tendency of ordering of the dislocation distribution and the reduction in the dislocation density might be expressed as Equation (15), corresponding to the minimization of the elastic energy. Alternatively, it corresponds to the minimization of the rate of plastic work under the external stress, as in Equation (16), due to the reduced dislocation density and increased glide resistance. Although the evolution processes might correspond to the maximization of the rate of plastic work under the instantaneously fixed internal stress, the overall tendency is towards the equilibrium state with the minimum total energy dissipation rate, as expressed in Equation (17), which seems to coincide with the MinEPP proposal of Prigogine [33]. This is the second dominant mechanism. As Equations (14) and (17) signify, the two dominant mechanisms correspond to the maximization and minimization of the total energy dissipation rate, respectively, which agrees with our previous findings in other complex systems [34], as well.

(15) $$\max\frac{{\dot{N}}_{w}-{\dot{N}}_{b}}{\dot{N}}=\frac{dN_{w}-dN_{b}}{dN}$$

(16) $$\min{\dot{W}}_{p}=-\frac{4}{3}\lambda_{1}\tau \varphi$$

(17) minΣ

It is interesting to mention that the fourth-power law [35,36] might be related with the first dominant mechanism. This law relates the stress jump (Δ*σ*) through a steady shock wave and the maximum strain rate (${\dot{\varepsilon }}_{\max}$) through a fourth power, as in Equation (18), where *s*_1_ and *c*_0_ are material constants, *A* is an assumed invariant, *ρ*_0_ is the initial mass density, and *O*(*x*) means the term of the same order as the involved expression *x*. It is further revealed that *A* has dimensions of a specific action [36], and might be taken as a dissipative action [37]. If the principle of least action [38] works here, the invariance of *A* might actually mean its minimization. According to Equations (19) and (20) [35,36] (where δ*E* is the dissipated energy, d*t* is the rise time of the wave, and *ε*_H_ is the Hugoniot strain), δ*E* is constant for a specific shock. Therefore, the minimization of *A* might correspond to the minimization of d*t*, and the maximization of δ*E*/d*t*, i.e., the maximization of the energy dissipation rate.

(18) $${\dot{\varepsilon }}_{\max}=\frac{s_{1}}{3A{\left(\rho_{0}c_{0}^{2}\right)}^{3}}{\left(\Delta \sigma \right)}^{4}+O\left({\left(\Delta \sigma \right)}^{5}\right)$$

*A* = δ*E* d*t* (19)

δ*E* = 1/3*ρ*_0_ *c*_0_^2^ *s*_1_ *ε*_H_^3^ + *O*(*ε*_H_^4^) (20)

When the first dominant mechanism solely works, the plastic shearing rate maximizes under fixed *τ* and *λ*_1_. According to the Orowan relation, as described in the preceding section, the dislocation density and the dislocation speed will maximize. The maximization of the dislocation speed and the compatibility of deformation [39] homogenize the dislocation distribution.

When the second dominant mechanism solely works, the dislocation density tends to decrease (the global minimum is zero), and the dislocation distribution tends to heterogenize, enhancing the difference of the dislocation density between the two phases in Figure 2.

When the two dominant mechanisms co-work, they compromise through competition. The dislocation density can reach neither the minimum nor the maximum. The dislocation distribution can reach neither the optimal configuration with minimum elastic energy nor the random configuration with high elastic energy. Maximization of the dislocation speed hinders the ordering of the dislocation distribution. Meanwhile, as indicated in Equations (1)–(11), under either thermal activation or phonon drag, the increase in the dislocation density will reduce the dislocation speed.

**Figure 3:**
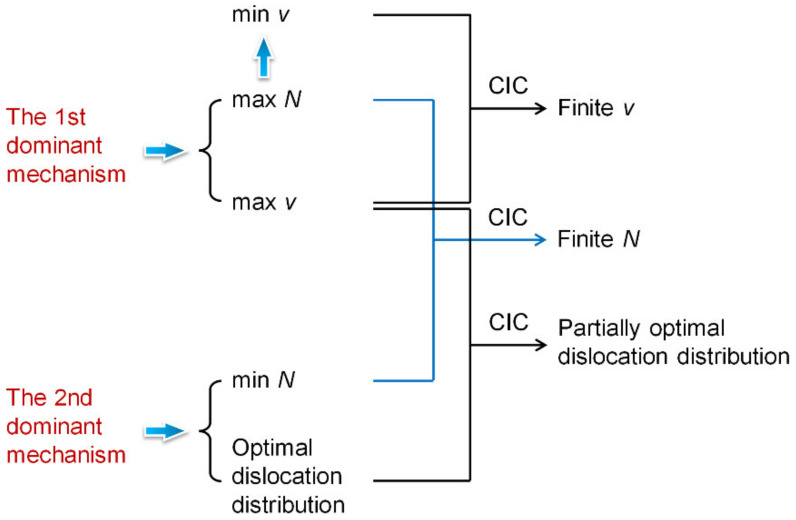
Compromise in competition between the two dominant mechanisms.

The above descriptions are illustrated in Figure 3. It is apparent that the realization of both the two dominant mechanisms is based on dislocation behaviors, and the compromise in competition between these two dominant mechanisms is also reflected in their effects on the dislocation density, distribution, and speed.

## 5. Applications to Constitutive Models

Based on the dominant mechanisms, under the mesoscience framework, we can establish the stability condition and thus the mesoscale model for the steady state, which can be developed further to describe the dynamic states through proper routes, e.g., applying it to each computational grid of a dynamic model. Another convenient route might be adopting the dominant mechanisms directly in the dynamic constitutive models or establishing the stability condition to improve the existent constitutive models.

The first dominant mechanism has actually been employed in several constitutive models though not through its extremal form. For example, Ziegler’s MaxEPP, as a stricter form of this dominant mechanism, has been used in [31,32,40]. The fourth-power law, seemingly related with this dominant mechanism, has also been employed in [41] and possibly in the PTW model for the overdriven-shock regime [13].

The second dominant mechanism has been adopted partially through the equations describing the evolution of dislocation density (not through the extremal form either), e.g., in [2,4,31]. However, a fixed volume fraction of cell blocks has been adopted in [31], and the recovery rate is assumed to be zero in [2], which might be possible points to further improve the models.

Since these two dominant mechanisms co-work in the mesoregime, it is better to take into account both of them simultaneously and completely, i.e., to incorporate their CIC in an extremal form. Here, we propose a scheme of employing these two dominant mechanisms to account for the heterogeneous distribution of dislocations. We model the heterogeneous dislocation distribution as a two-phase structure: A dense phase with high dislocation density corresponding to the cell walls, and a dilute phase with low dislocation density corresponding to the cell block, as illustrated in Figure 2. The mass fraction of the dense phase is *f*_w_. The proposed scheme will be applied to the following three increasingly complicated constitutive models, and exemplified with 6061-T6 Al alloy under shock magnitudes of 2.1, 3.7, and 9.0 GPa to facilitate the comparison with the existing experimental data.

### 5.1. Application to Model #1

For each phase, we adopt the constitutive model of Molinari and Ravichandran [22] for the uniaxial strain conditions, as expressed in Equations (21)–(46), where (and hereinafter) in the subscripts *i* = w, b, and “HEL” mean the Hugoniot elastic limit at the rear of the elastic precursor, which is the initial state here. The meanings of the quantities not introduced before are given in Table 1 and Table 2, where the shock stress magnitude *σ*_1,–_ = −*T*_1,–_ for the present compression cases (the subscript “–” means the final Hugoniot state).

(21) $$\frac{d\lambda_{1,p,i}}{d\xi }=\frac{2}{3C}\lambda_{1,p,i}\varphi_{i}$$

(22) $$\varphi_{i}=bN_{m,i}v_{i}$$

(23) $$N_{m,i}=N_{m,\mathrm{HEL},i}\left(1+\frac{\alpha_{b}\gamma_{p,i}}{bN_{\mathrm{HEL},i}}\right)\exp\left(-\alpha_{t}\alpha_{b}\gamma_{p,i}\right)$$

(24) $$\gamma_{p,i}=-\frac{3}{2}\ln\lambda_{1,p,i}$$

(25) $$v_{i}=c_{1}{\left(\frac{\left|\tau_{i}+\tau_{a,i}\right|}{T_{1}^{*}}\right)}^{M}$$

(26) $$\tau_{i}=\frac{1}{2}\left(T_{1,i}-\frac{T_{2,i}}{\lambda_{1,i}}\right)$$

(27) $$\tau_{a,i}=\tau_{a0}\left[1+{\left(\frac{\gamma_{p,i}}{\gamma_{0}}\right)}^{1/n}\right]$$

(28) $$T_{1,i}=\rho_{0}\frac{F_{1,i}}{\lambda_{1,p,i}}$$

(29) $$T_{2,i}=\rho_{0}F_{2,i}\sqrt{\lambda_{1,p,i}}$$

(30) $$\lambda_{1,i}=\left(\varepsilon_{1,e,i}+1\right)\lambda_{1,p,i}$$

(31) $$F_{1,i}=a_{1}+A_{1}\varepsilon_{1,e,i}+A_{2}\varepsilon_{1,e,i}^{2}+B_{1}\varepsilon_{2,e,i}+B_{2}\varepsilon_{2,e,i}^{2}+D\varepsilon_{1,e,i}\varepsilon_{2,e,i}$$

(32) $$F_{2,i}=a_{1n}+A_{1n}\varepsilon_{1,e,i}+A_{2n}\varepsilon_{1,e,i}^{2}+B_{1n}\varepsilon_{2,e,i}+B_{2n}\varepsilon_{2,e,i}^{2}+D_{n}\varepsilon_{1,e,i}\varepsilon_{2,e,i}$$

(33) $$\varepsilon_{1,e,i}=\frac{-B_{i}+\sqrt{B_{i}^{2}-4A_{2}G_{i}}}{2A_{2}}$$

(34) $$\varepsilon_{2,e,i}=\sqrt{\lambda_{1,p,i}}-1$$

(35) $$B_{i}=A_{1}+D\varepsilon_{2,e,i}-C^{2}{\left(1+\varepsilon_{2,e,i}\right)}^{4}$$

(36) $$G_{i}=B_{1}\varepsilon_{2,e,i}+B_{2}\varepsilon_{2,e,i}^{2}+\left(C^{2}\lambda_{1,\mathrm{HEL}}-\frac{T_{1,\mathrm{HEL}}}{\rho_{0}}\right){\left(1+\varepsilon_{2,e,i}\right)}^{2}-C^{2}{\left(1+\varepsilon_{2,e,i}\right)}^{4}$$

(37) $$A_{1}=2a_{2}$$

(38) $$A_{2}=3a_{4}$$

(39) $$B_{1}=4a_{2}+2a_{3}$$

(40) $$B_{2}=12a_{4}+5a_{5}+a_{6}$$

(41) $$D=12a_{4}+4a_{5}$$

(42) $$A_{1n}=2a_{2}+a_{3}$$

(43) $$A_{2n}=3a_{4}+a_{5}$$

(44) $$B_{1n}=4a_{2}+a_{3}$$

(45) $$B_{2n}=12a_{4}+3a_{5}$$

(46) $$D_{n}=12a_{4}+5a_{5}+a_{6}$$

The adopted values of relevant parameters are listed in Table 2 for 6061-T6 Al alloy [22]. The values of *λ*_1,HEL_ and *T*_1,HEL_ are obtained by solving Equation (47) with *λ*_1,p,HEL_ = 1, using the Newton-Raphson method. The value of *u*_p,HEL_ is calculated accordingly via Equations (48) and (49), where *C*_e_ is the speed of the elastic precursor. The *C* values in Table 2 are calculated with the Rayleigh-line constraint, i.e., Equation (50), where *T*_1,–_ is assigned (through *σ*_1,–_ = 2.1, 3.7, and 9.0 GPa, respectively) and *λ*_1,–_ is calculated via the yield condition, i.e., Equation (51), along with Equations (26)–(28).

(47) $$\frac{1}{2}\left(T_{1,\mathrm{HEL}}-\frac{T_{2,\mathrm{HEL}}}{\lambda_{1,\mathrm{HEL}}}\right)+\tau_{a0}=0$$

(48) $$u_{p,\mathrm{HEL}}=\left(1-\lambda_{1,\mathrm{HEL}}\right)C_{e}$$

(49) $$C_{e}=\sqrt{\frac{-T_{1,\mathrm{HEL}}}{\left(1-\lambda_{1,\mathrm{HEL}}\right)\rho_{0}}}$$

(50) $$C=\sqrt{\frac{T_{1,-}-T_{1,\mathrm{HEL}}}{\left(\lambda_{1,-}-\lambda_{1,\mathrm{HEL}}\right)\rho_{0}}}$$

(51) $$\tau_{-}+\tau_{a,-}=0$$

The values of *N*_m,HEL,w_ and *N*_HEL,w_ in Table 2 are taken from [22], and those of *N*_m,HEL,b_ and *N*_HEL,b_ have been set to be around 2% smaller (some other values have been tested as well, and the results are qualitatively consistent), reflecting reasonably the spatial fluctuation of the dislocation density. These are the only differences assumed between the two phases. Subsequently, each phase evolves through the integration of Equation (21) from the initial HEL state to the final Hugoniot state, via the fourth-order Runge-Kutta method. The mass fraction *f*_w_ evolves as well, and the profiles of all average quantities can be achieved. For instance, the average particle speed *u*_p_ can be calculated using Equation (52), based on Equation (53).

(52) $$u_{p}=f_{w}u_{p,w}+\left(1-f_{w}\right)u_{p,b}$$

(53) $$u_{p,i}=u_{p,\mathrm{HEL}}-\left(\lambda_{1,i}-\lambda_{1,\mathrm{HEL}}\right)C$$

Due to the introduction of the variable *f*_w_, the above model is not closed anymore. Therefore, to determine *f*_w_, additional constraints are needed. We will use the dominant mechanisms to build the stability condition as an additional constraint.

As proposed above, for such a two-phase system, the first dominant mechanism can be expressed as Equation (54) along with Equation (55), and the second one can be expressed as Equation (56) supported by Equation (57). The CIC between these two dominant mechanisms can be expressed as Equation (58). To transform this bi-objective optimization into a single-objective one, we adopt our previous technique [42], and the resultant stability condition is shown in Equation (59), where *m_j_*_,min_ and *m_j_*_,max_ (*j* = 1, 2) mean the minimum and maximum of *m_j_*, respectively.

(54) $$\min m_{1}=\frac{{\dot{N}}_{w}-{\dot{N}}_{b}}{\dot{N}}=\frac{dN_{w}-dN_{b}}{dN}$$

(55) $$N=f_{w}N_{w}+\left(1-f_{w}\right)N_{b}$$

(56) $$\min m_{2}={\dot{W}}_{p}=f_{w}{\dot{W}}_{p,w}+\left(1-f_{w}\right){\dot{W}}_{p,b}$$

(57) $${\dot{W}}_{p,i}=-\frac{4}{3}\lambda_{1,i}\tau_{i}\varphi_{i}$$

(58) $$\min\left(\begin{array}{c} m_{1}\\ m_{2} \end{array}\right)$$

(59) $$\min\left(\frac{m_{1}-m_{1,\min}}{m_{1,\max}-m_{1,\min}}+\frac{m_{2}-m_{2,\min}}{m_{2,\max}-m_{2,\min}}\right)$$

**Figure 4:**
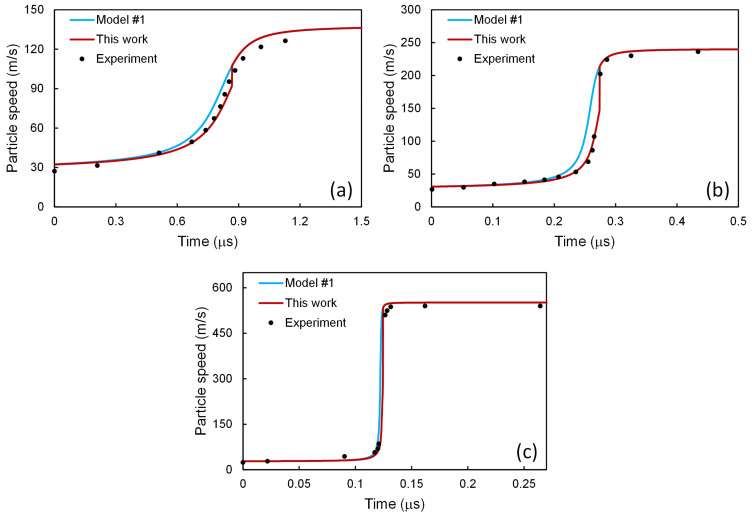
Particle speed profiles for 6061-T6 Al alloy at shock stress amplitudes of (**a**) 2.1, (**b**) 3.7, and (**c**) 9.0 GPa. The present scheme has been applied to model #1 [22], and the results are compared with those from model #1 [22] and the experimental ones [43].

Along with such a stability condition (Equation (59)), the two-phase model is closed, and all the quantities can be determined along the profile. The particle speed profiles thus obtained are presented in Figure 4 for 6061-T6 Al alloy at shock stress amplitudes *σ*_1,–_ = 2.1, 3.7, and 9.0 GPa, in comparison with those calculated from the original model of Molinari and Ravichandran [22], and the experimental results of Johnson and Barker [43]. It is interesting to note that rather than the single-phase assumption in the original model of Molinari and Ravichandran [22], the present model considers a two-phase distribution where the second phase is introduced via a negligible change in the initial dislocation density. Such a simple modification has brought the profiles closer to the experimental ones. If more sophisticated schemes are adopted, e.g., considering the interphase interactions, more improvements might be expected.

### 5.2. Application to Model #2

The above constitutive model of Molinari and Ravichandran [22] contains only one ordinary differential equation (Equation (21)) for each phase, and the detailed evolution of dislocation density has not been accounted for. In this application, we adopt the descriptions of dislocation evolution in the literature [2]. Accordingly, in addition to Equation (21), two more ordinary differential equations have been introduced, i.e., Equations (60) and (61), to replace Equation (23), and supporting equations are given in Equations (62)–(70), where *N*_im_ is the immobile dislocation density, subscripts “nuc”, “multi”, “ann”, and “trap” mean the nucleation, multiplication, annihilation, and trapping of dislocations, respectively, and *f*_het_ is the probability distribution function for heterogeneous nucleation. The definitions and values of other relevant parameters are listed in Table 3. Since *τ* < 0 for compression in the present work, its absolute value is adopted. Homogeneous nucleation cannot occur within the present range of shock stresses [4], so only heterogeneous nucleation has been taken into account in Equation (64). The data in Table 2 excluding those specifically for Equation (23) (*N*_m,HEL,*i*_, *α*_b_, *α*_t_) are employed, as well.

(60) $$\frac{dN_{m,i}}{d\xi }=-\frac{1}{C}{\dot{N}}_{m,i}$$

(61) $$\frac{dN_{\mathrm{im},i}}{d\xi }=-\frac{1}{C}{\dot{N}}_{\mathrm{im},i}$$

(62) $${\dot{N}}_{m,i}={\dot{N}}_{\mathrm{nuc},i}+{\dot{N}}_{\mathrm{multi},i}-{\dot{N}}_{\mathrm{ann},i}-{\dot{N}}_{\mathrm{trap},i}$$

(63) $${\dot{N}}_{\mathrm{im},i}={\dot{N}}_{\mathrm{trap},i}$$

(64) $${\dot{N}}_{\mathrm{nuc},i}=\left\{\begin{array}{cc} \alpha_{\mathrm{het}}f_{\mathrm{het},i}\left|{\dot{\tau }}_{i}\right| & {\dot{\tau }}_{i}<0\\ 0 & \mathrm{otherwise} \end{array}\right.$$

(65) $${\dot{N}}_{\mathrm{multi},i}=\frac{\delta }{b}\varphi_{i}$$

(66) $${\dot{N}}_{\mathrm{ann},i}=\alpha_{\mathrm{ann}}N_{m,i}\varphi_{i}$$

(67) $${\dot{N}}_{\mathrm{trap},i}=\frac{\varphi_{i}}{b}\left[\alpha_{\mathrm{dis}}\sqrt{N_{i}}+\frac{\alpha_{p}\pi d_{p}}{{\left(\lambda_{p}+d_{p}\right)}^{2}}+\frac{1}{d}\right]$$

(68) $$f_{\mathrm{het},i}=\left\{\begin{array}{cc} \frac{m+1}{{\left(\tau_{b}-\tau_{a}\right)}^{m+1}}{\left(\left|\tau_{i}\right|-\tau_{a}\right)}^{m} & \tau_{a}<\left|\tau_{i}\right|\leq \tau_{b}\\ 0 & \mathrm{otherwise} \end{array}\right.$$

(69) $$N_{m,\mathrm{HEL},i}=f_{\mathrm{HEL}}N_{\mathrm{HEL},i}$$

(70) $$N_{\mathrm{im},\mathrm{HEL},i}=\left(1-f_{\mathrm{HEL}}\right)N_{\mathrm{HEL},i}$$

**Figure 5:**
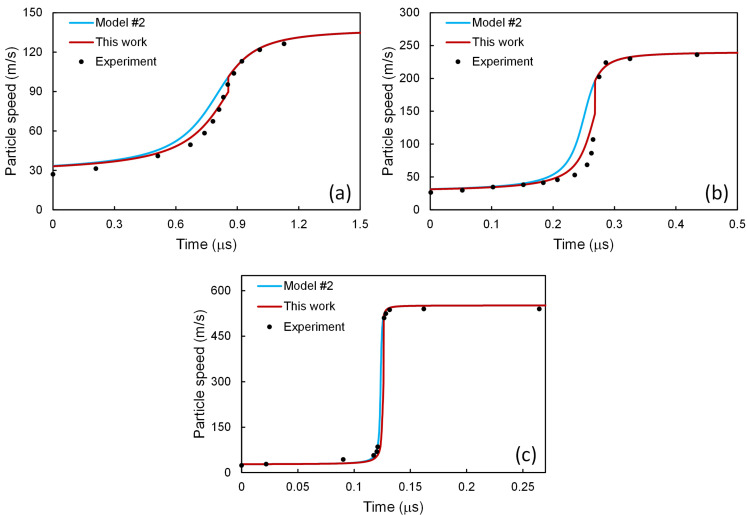
Particle speed profiles for 6061-T6 Al alloy at shock stress amplitudes of (**a**) 2.1, (**b**) 3.7, and (**c**) 9.0 GPa. The present scheme has been applied to model #2 [2,22], and the results are compared with those from model #2 [2,22] and the experimental ones [43].

Along with Equations (47)–(59), the whole has been solved, and the results have been presented in Figure 5. It is observed that in this model considering detailed dislocation processes, involving the CIC of the two dominant mechanisms to account for the heterogeneous distribution of dislocations can improve the agreement of the calculated profiles with the experimental results, as well.

### 5.3. Application to Model #3

**Figure 6:**
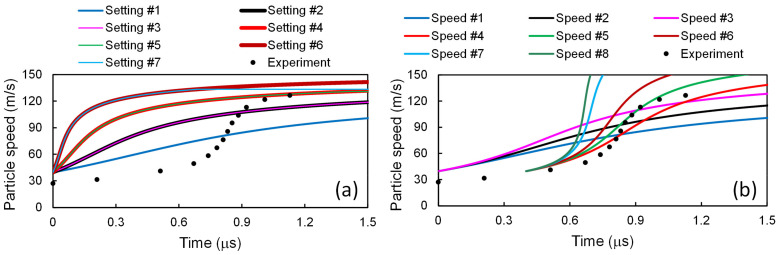
Particle speed profiles for 6061-T6 Al alloy at shock stress amplitude of 2.1 GPa, calculated with the model of Austin and McDowell [2], (**a**) with *C* = 5353 m/s, where setting #1 means *α*_0_ = 1.0, *β*_0_ = 0.1, setting #2 means *α*_0_ = 0.9, *β*_0_ = 0.1, setting #3 means *α*_0_ = 0.9, *β*_0_ = 0.2, setting #4 means *α*_0_ = 0.8, *β*_0_ = 0.1, setting #5 means *α*_0_ = 0.8, *β*_0_ = 0.2, setting #6 means *α*_0_ = 0.7, *β*_0_ = 0.1, setting #7 means *α*_0_ = 0.7, *β*_0_ = 0.2, and (**b**) with *α*_0_ = 1.0, *β*_0_ = 0.1, where speed #1 means *C* = 5353 m/s, speed #2 means *C* = 5383 m/s, speed #3 means *C* = 5407 m/s, speed #4 means *C* = 5437 m/s, speed #5 means *C* = 5457 m/s, speed #6 means *C* = 5477 m/s, speed #7 means *C* = 5519 m/s, speed #8 means *C* = 5535 m/s. The experimental results [43] have been presented for comparison.

The present scheme has been applied further to the complete model of Austin and McDowell [2]. In comparison with the above model #2, the dislocation speed in Equation (25) has been replaced with Equation (71), and supporting expressions have been given in Equations (72)–(80). Since the temperature increase has been involved, Equation (81) is adopted to account for the contribution from the thermoelastic effect, and an additional ordinary differential equation is adopted to account for the contribution from the plastic work, as in Equation (82). The definitions and values of relevant parameters have been given in Table 4. The profile of the particle speed is sensitive to *α*_0_ though insensitive to *β*_0_ when *α*_0_ is high, as illustrated in Figure 6a. The values of *α*_0_ and *β*_0_ proposed by Austin and McDowell [4] have been employed, and the other values in Table 4 are all taken from Austin and McDowell [2] (the *α*_0_ and *β*_0_ values therein do not work well) if not described otherwise. The profile is sensitive to *C* as well, as shown in Figure 6b. The *C* values in Table 2 are obtained with Equation (50), but not all of them result in satisfactory agreement with the experimental profiles under the setting of other parameters in Table 4. Therefore, some *C* values have been modified a little as Austin and McDowell did [2], based on the empirical equation of state, as in Equation (83), and the adopted ones have also been listed in Table 4. According to the model of Austin and McDowell [2], the initial state is at the onset of the main plastic wave, denoted with a subscript “+” in Table 4, rather than the HEL state, and relevant values have been calculated using the parameter values of Austin and McDowell [2] and given in Table 4 as well, corresponding to Equations (84)–(86). The value of *N*_+,b_ is set a little (about 5%) smaller than that [2] of *N*_+,w_. Since a certain extent of work hardening should occur in the plastic precursor, the value of *N*_+,w_ (corresponding to the “+” state at the rear of the plastic precursor) in Table 4 is set to be much higher than that (taken from [22] and actually based on experimental results [43]) of *N*_HEL,w_ (corresponding to the HEL state at the rear of the elastic precursor) in Table 2, based on reasonable calculations [2]. However, the value of *N*_m,HEL,w_ in Table 2 or from Equation (69) is close to that of *N*_m,+,w_ from Equation (84), indicating the large increase in the immobile dislocation density in the plastic precursor. The values of *b*_1_–*b*_3_ in Table 4 are taken from the article of Molinari and Ravichandran [22].

(71) $$v_{i}=\frac{c_{s,i}h_{i}}{\left[\exp\left(\frac{\Delta G_{i}}{k\theta_{i}}\right)-1\right]\frac{c_{s,i}h_{i}}{\chi \nu_{D}b}+1}$$

(72) $$c_{s,i}=\sqrt{\frac{\mu_{i}}{\rho_{i}}}$$

(73) $$\mu_{i}=\mu_{0}\left[1+\frac{1}{\mu_{0}}\frac{\partial \mu }{\partial p}\left(p_{i}-p_{0}\right){\left(\frac{V_{i}}{V_{0}}\right)}^{1/3}+\frac{1}{\mu_{0}}\frac{\partial \mu }{\partial \theta }\left(\theta_{i}-\theta_{0}\right)\right]$$

(74) $$\theta_{i}=\theta_{0}+\Delta \theta_{e,i}+\Delta \theta_{p,i}$$

(75) $$h_{i}=\sqrt{\zeta_{i}^{2}+1}-\zeta_{i}$$

(76) $$\zeta_{i}=\frac{B_{0,i}c_{s,i}}{2b\sqrt{\tau_{i}^{2}-\tau_{\mu ,i}^{2}}}$$

(77) $$\tau_{\mu ,i}=\frac{\mu_{i}}{\mu_{0}}\left(\beta_{0}\tau_{\mathrm{dis},0,i}+\tau_{p,0}\right)$$

(78) $$\Delta G_{i}=g_{0}\mu_{i}b^{3}{\left[1-{\left(\frac{\tau_{i}}{\tau_{\mathrm{dis},0,i}+\tau_{p,0}}\right)}^{q}\right]}^{r}$$

(79) $$\tau_{\mathrm{dis},0,i}=\alpha_{0}\mu_{0}b\sqrt{N_{i}}$$

(80) $$\tau_{p,0}=\beta_{p}\frac{\mu_{0}b}{\lambda_{p}}$$

(81) $$\Delta \theta_{e,i}=b_{1}\left(\varepsilon_{1,e,i}+2\varepsilon_{2,e,i}\right)+b_{2}{\left(\varepsilon_{1,e,i}+2\varepsilon_{2,e,i}\right)}^{2}+b_{3}\left(\varepsilon_{1,e,i}+2\varepsilon_{2,e,i}\right)\varepsilon_{2,e,i}$$

(82) $$\frac{d\left(\Delta \theta_{p,i}\right)}{d\xi }=\frac{4\beta }{3\rho_{0}c_{\eta }C}\lambda_{1,i}\tau_{i}\varphi_{i}$$

(83) $$C=c_{0}+s_{1}u_{p,-}$$

(84) $$N_{m,+,i}=f_{+}N_{+,i}$$

(85) $$N_{\mathrm{im},+,i}=\left(1-f_{+}\right)N_{+,i}$$

(86) $$u_{p,i}=u_{p,+}-\left(\lambda_{1,i}-\lambda_{1,+}\right)C$$

**Figure 7:**
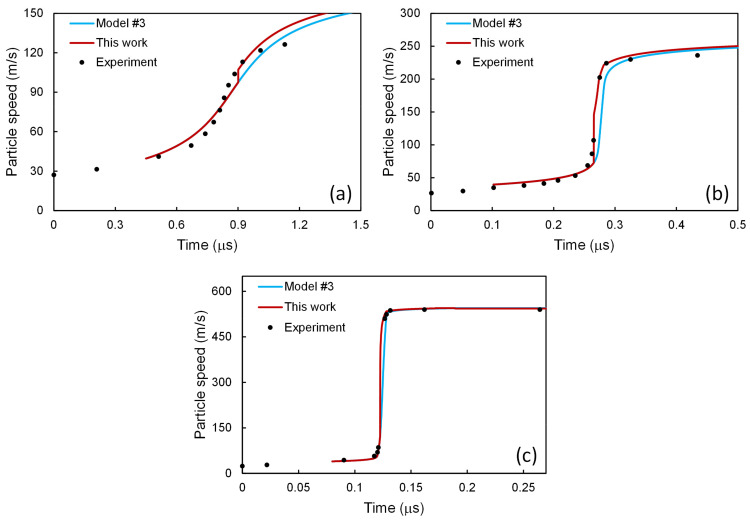
Particle speed profiles for 6061-T6 Al alloy at shock stress amplitudes of (**a**) 2.1, (**b**) 3.7, and (**c**) 9.0 GPa. The present scheme has been applied to model #3 [2,4], and the results are compared with those from model #3 [2,4] and the experimental ones [43].

The particle speeds obtained via integrating from the “+” state to the “−” state are given in Figure 7. Since the “+” state is different from the HEL state, the profiles here do not cover the first several experimental points, as in the results of Austin and McDowell [2]. It is observed that considering the two-phase structure and introducing the stability condition of Equation (59), can improve the agreement of this model with the experiments, as well.

### 5.4. Summary

**Figure 8:**
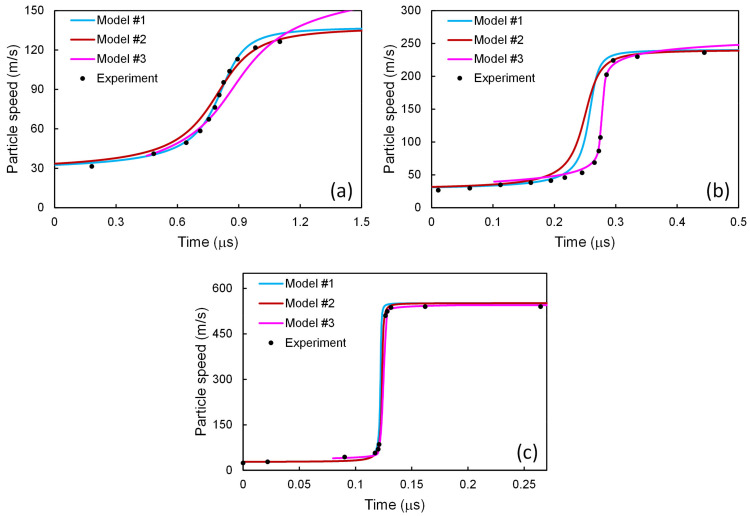
Particle speed profiles for 6061-T6 Al alloy at shock stress amplitudes of (**a**) 2.1, (**b**) 3.7, and (**c**) 9.0 GPa. The results of the three models [2,4,22] are compared with the experimental ones [43].

The results of the above three models have been put together in Figure 8. Among these three models, as shown in Figure 8a, for *σ*_1,–_ = 2.1 GPa, the results calculated with the model of Molinari and Ravichandran [22] seems to agree best with the experimental ones. For *σ*_1,–_ = 3.7 GPa, as in Figure 8b, the results from the model of Austin and McDowell [2] seems to agree best with the experimental ones. However, for *σ*_1,–_ = 9.0 GPa, as in Figure 8c, the best results come from the model of Molinari and Ravichandran [22], combined with the dislocation processes taken from the model of Austin and McDowell [2]. In other words, none of the above three models can reproduce the experimental results satisfactorily for all the three shock stress amplitudes. In contrast, via accounting for the two-phase heterogeneity and introducing the stability condition, the present work improves the agreement for all cases, as shown in Figure 4 Figure 5 and Figure 7.

It is necessary to note that although the first and the second dominant mechanisms have been given three possible expressions each as in Equations (12)–(14) and Equations (15)–(17), respectively, we actually adopted Equations (12) and (16) in the above applications. The reason lies in three aspects. Firstly, it is apparent that to build a computable CIC combination, the two dominant mechanisms should not be expressed with the same quantity. Secondly, since the calculation of Σ is not straightforward, Equations (14) and (17) have been abandoned here. Thirdly, the combination of Equations (13) and (15) results in only trivial solutions in the above applications, which should be discarded.

## 6. Conclusions

In this work, we have elucidated the mesoscale complexity in the viscoplastic deformation of metals, under the framework of mesoscience. Based on analyzing the processes that determine the dislocation density and distributions, two competing dominant mechanisms have been revealed, i.e., maximization of the rate of plastic work, and minimization of the elastic energy, which lead to extreme results individually, but produce complexity when co-working.

Mathematical expressions have been proposed to reflect the dominant mechanisms, and subsequently a stability condition has been established as the CIC between them. Such a stability condition has been further used to close two-phase models, and the results have shown improvement over the original constitutive models. In the near future, the present scheme will be adopted to develop more sophisticated models, through improved routes of applying the stability condition, and more advantages are expected to emerge.

## Figures and Tables

**Table 1:** Definitions of relevant quantities in Equations (21)–(46) [22].

Quantity	Definition
*λ*_1,p_	longitudinal plastic stretch
*ξ*	coordinate moving with *C*
*N*_m_	mobile dislocation density
*γ*_p_	plastic shear strain
*τ*_a_	yield shear stress
*T*_1_	longitudinal Piola-Kirchhoff stress
*T*_2_	transverse Piola-Kirchhoff stress
*F*_1_	function for elastic deformation
*F*_2_	function for elastic deformation
*ε*_1,e_	longitudinal elastic strain
*ε*_2,e_	transverse elastic strain
*A*_1_, *A*_2_, *B*_1_, *B*_2_, *D*	intermediate parameters
*A*_1n_, *A*_2n_, *B*_1n_, *B*_2n_, *D*_n_	intermediate parameters
*B*, *G*	intermediate parameters
*u*_p_	particle speed

**Table 2:** Material and operating parameters in Equations (21)–(46) and their values for 6061-T6 Al alloy at ambient conditions (taken from [22] unless specified otherwise).

Parameter	Value	Unit	Definition
*ρ*_0_	2703	kg/m^3^	initial density
*b*	2.86 × 10^−10^	m	Burgers’ vector magnitude
*a*_1_	0	m^2^/s^2^	elastic constant
*a*_1n_	0	m^2^/s^2^	elastic constant
*a*_2_	2.028 × 10^7^	m^2^/s^2^	elastic constant
*a*_3_	−2.044 × 10^7^	m^2^/s^2^	elastic constant
*a*_4_	−6.64 × 10^7^	m^2^/s^2^	elastic constant
*a*_5_	1.575 × 10^8^	m^2^/s^2^	elastic constant
*a*_6_	−1.428 × 10^8^	m^2^/s^2^	elastic constant
*c*_1_	0.168	m/s	fitting parameter
$T_{1}^{*}$	1.6	MPa	fitting parameter
*M*	1.78		inverse of the strain rate sensitivity
*τ*_a0_	120	MPa	initial back stress
*γ*_0_	0.52		reference strain
*n*	1.55		hardening parameter
*N*_m,HEL,w_	8.18 × 10^12^	1/m^2^	initial mobile dislocation density in the cell wall
*N*_m,HEL,b_	8.00 × 10^12^	1/m^2^	initial mobile dislocation density in the cell block
*α*_b_	3.5 × 10^5^	1/m	breeding coefficient
*α*_t_	0		trapping coefficient
*N*_HEL,w_	8.18 × 10^12^	1/m^2^	initial total dislocation density in the cell wall
*N*_HEL,b_	8.00 × 10^12^	1/m^2^	initial total dislocation density in the cell block
*λ*_1,HEL_	0.995758		HEL longitudinal stretch
*T*_1,HEL_	−475	MPa	HEL longitudinal Piola-Kirchhoff stress
*C*	5457.3146	m/s	shock wave speed at *σ*_1,–_ = 2.1 GPa
	5600.2774		shock wave speed at *σ*_1,–_ = 3.7 GPa
	6018.3672		shock wave speed at *σ*_1,–_ = 9.0 GPa
*u*_p,HEL_	27.295879	m/s	HEL particle speed

**Table 3:** Parameters in Equations (64)–(70) and their values for 6061-T6 Al alloy at ambient conditions [2,22].

Parameter	Value	Unit	Definition
*α*_het_	7.4 × 10^13^	1/m^2^	heterogeneous nucleation coefficient
*α*_ann_	0.5		annihilation coefficient
*α*_dis_	0.015		network trapping coefficient
*α*_p_	0.02		precipitate trapping coefficient
*d*_p_	1.0 × 10^−8^	m	precipitate size
*λ*_p_	7.0 × 10^−8^	m	precipitate spacing
*d*	4.0 × 10^−5^	m	mean grain size
*m*	1.0		shape constant
*f*_HEL_	1.0		initial fraction of mobile dislocations
*δ*	3.5 × 10^5^	1/m	multiplication coefficient
*τ*_a_	106	MPa	lower-bound shear stress for heterogeneous nucleation
*τ*_b_	920	MPa	upper-bound shear stress for heterogeneous nucleation

**Table 4:** Material and operating parameters in Equations (71)–(86) and their values for 6061-T6 Al alloy at ambient conditions (taken from [2,4] unless described otherwise).

Parameter	Value	Unit	Definition
*k*	1.380649 × 10^−23^	J/K	Boltzmann’s constant
*χ*	0.05		scaling parameter
*ν*_D_	8.0 × 10^12^	1/s	Debye’s frequency
*g*_0_	0.65		thermal activation parameter
*q*	0.5		thermal activation parameter
*r*	2.0		thermal activation parameter
*μ*_0_	27.627	GPa	initial shear modulus
1/*μ*_0_ (∂*μ*/∂*p*)	65 × 10^−3^	1/GPa	pressure coefficient of shear modulus
1/*μ*_0_ (∂*μ*/∂*θ*)	−0.62 × 10^−3^	1/K	temperature coefficient of shear modulus
*θ*_0_	300.0	K	initial temperature
*p*_0_	0.0	Pa	initial pressure
*z*	4.0		number of atoms per unit cell
*α*_0_	1.0		dislocation interaction coefficient
*β*_0_	0.0		long-range interaction factor
*β*_p_	0.84		Orowan looping factor
*C*	5457.3146	m/s	shock wave speed at *σ*_1,–_ = 2.1 GPa
	5606.2939		shock wave speed at *σ*_1,–_ = 3.7 GPa
	6011.1869		shock wave speed at *σ*_1,–_ = 9.0 GPa
*c*_0_	5350.0	m/s	material constant
*s*_1_	1.34		material constant
*N*_+,w_	2.0 × 10^14^	1/m^2^	initial total dislocation density in the cell wall
*N*_+,b_	1.9 × 10^14^	1/m^2^	initial total dislocation density in the cell block
*f*_+_	0.006		initial fraction of mobile dislocations
*λ*_1,+_	0.993711		initial longitudinal stretch
*T*_1,+_	−675	MPa	HEL longitudinal Piola-Kirchhoff stress
*λ*_1,p,+_	0.9994002		initial longitudinal plastic stretch
*u*_p,+_	39.629214	m/s	initial particle speed
*b*_1_	−593	K	thermomechanical parameter
*b*_2_	−130	K	thermomechanical parameter
*b*_3_	1350	K	thermomechanical parameter
*β*	1.0		Taylor-Quinney coefficient
*c_η_*	880.0	J/(g K)	specific heat at constant configuration

## Data Availability

Data sharing is not applicable to this article.
